# Nutrition Info and Other Front-of-Package Labels and Simulated Food and Beverage Purchases: A Randomized Clinical Trial

**DOI:** 10.1001/jamanetworkopen.2025.37389

**Published:** 2025-10-17

**Authors:** Anna H. Grummon, Kevin O’Sullivan, Joshua Petimar, Cristina J. Y. Lee, Amanda B. Zeitlin, Lauren P. Cleveland, Caroline Collis, Aviva A. Musicus, Verena Tiefenbeck, Elgar Fleisch, Jason P. Block

**Affiliations:** 1 Department of Pediatrics, Stanford University School of Medicine, Palo Alto, California; 2 Department of Health Policy, Stanford University School of Medicine, Stanford, California; 3 Department of Management, Technology, and Economics, ETH Zurich, Zurich, Switzerland; 4 Department of Population Medicine, Harvard Pilgrim Health Care Institute, Boston, Massachusetts; 5 Department of Nutrition, Gillings School of Global Public Health, University of North Carolina at Chapel Hill, Chapel Hill; 6 Center for Science in the Public Interest, Washington, DC; 7 Department of Nutrition, Harvard T. H. Chan School of Public Health, Boston, Massachusetts; 8 School of Business, Economics and Society, Friedrich-Alexander-Universität, Nürnberg, Germany; 9 Institute of Technology Management, University of St Gallen, St Gallen, Switzerland

## Abstract

**Importance:**

In January 2025, the US Food and Drug Administration (FDA) released a proposed rule to require front-of-package nutrition information (Nutrition Info) labels that would signal when packaged foods contain low, medium, or high levels of saturated fat, sodium, or added sugars (ie, nutrients of concern). However, it is unknown whether this labeling system would encourage healthier food and beverage purchases.

**Objectives:**

To test whether Nutrition Info labels similar to those proposed by the FDA could lead to healthier food and beverage purchases compared with other existing or proposed labeling systems.

**Design, Setting, and Participants:**

This randomized clinical trial was conducted online from October 31 to November 21, 2024. Participants included a national sample of US adults who reported being their household’s primary shopper. Data were analyzed from November 26, 2024, to August 27, 2025.

**Intervention:**

Participants were randomized to exposure to 1 of 6 front-of-package labeling systems: positive labels (ie, labels that communicate only the positive attributes of a food) only, Nutrition Info labels (similar to the FDA’s proposal), positive plus Nutrition Info labels, “High In” labels (similar to designs the FDA tested to signal when products contain high levels of nutrients of concern), positive plus High In labels, or spectrum labels (similar to designs used internationally rating products from least to most healthy).

**Main Outcomes and Measures:**

Participants shopped for foods and beverages in a large, simulated online grocery store. The primary outcome was healthfulness of participants’ food and beverage selections, assessed using the United Kingdom’s Ofcom Nutrient Profiling Model scores (ranging from 0 to 100, with higher scores indicating healthier choices). Scores were compared between groups using the average differential effect (ADE).

**Results:**

A total of 5636 participants completed the trial (3400 [60%] women; mean [SD] age, 40.3 [12.6] years). The spectrum labels led to healthier purchases compared with both the positive labels only (ADE, 2.42; 95% CI, 1.66-3.17; *P* < .001) and all other labeling systems (range of ADEs, −1.87 [95% CI, −2.63 to −1.11] to −2.45 [95% CI, −3.21 to −1.69]; *P* < .001). By contrast, the Nutrition Info, positive plus Nutrition Info, High In, and positive plus High In labels did not lead to healthier purchases compared with the positive labels only (range of ADEs, −0.04 [95% CI, −0.80 to 0.72; *P* = .92] to 0.54 [95% CI, −0.22 to 1.30; *P* = .16]). Label effects did not differ by nutrition literacy, household income, or educational attainment.

**Conclusions and Relevance:**

In this randomized clinical trial of food labeling systems, spectrum labels that rate foods from least to most healthy led to healthier purchases than positive labels and Nutrition Info labels similar to those proposed by the FDA. These findings suggest that spectrum labels may be more promising than both existing positive labels and the FDA’s proposed labels for promoting healthier food purchases.

**Trial Registration:**

ClinicalTrials.gov Identifier: NCT06516627

## Key Points

**Question:**

Could front-of-package nutrition information (Nutrition Info) labels proposed by the US Food and Drug Administration (FDA) help consumers buy healthier foods compared with other proposed or existing labels?

**Findings:**

In this randomized clinical trial with 5636 US adults, front-of-package labels did not lead participants to buy healthier foods compared with positive labels (ie, labels that communicate only the positive attributes of a food). By contrast, spectrum labels that rated foods from least to most healthy led participants to buy foods that were healthier than those with positive labels only and those with Nutrition Info labels.

**Meaning:**

These findings suggest that spectrum labels may promote healthier food purchases than the FDA’s proposed Nutrition Info labels.

## Teaser

This randomized clinical trial evaluated whether Nutrition Info labels similar to those proposed by the US Food and Drug Administration promote healthier food choices among participants shopping in a simulated online grocery store compared with other existing or proposed labeling systems.

## Introduction

Poor diet quality is responsible for approximately 500 000 deaths^1^ and more than $50 billion in health care costs in the US every year.^2^ While the causes of poor diet quality are complex, one important barrier to improving diet quality is that consumers often lack access to easy-to-understand nutrition information when deciding what foods to eat. The US requires most packaged foods to display the Nutrition Facts Label, but this label is displayed on the back or the side of product packaging and includes only numerical information that most consumers have difficulty understanding.^3,4^ Experts and policy-makers have therefore called for the US to require front-of-package food labels that interpret product healthfulness for consumers, rather than only providing numerical information.^5,6^ Many manufacturers and grocery chains have voluntarily implemented interpretative front-of-package and shelf-tag food labeling systems (eg, Guiding Stars, American Heart Association’s Heart-Check). However, these voluntary labeling systems typically communicate only the positive attributes of a food,^7^ despite evidence that labels are more effective when they also communicate a food’s negative attributes.^7,8,9^

To address these limitations, the US Food and Drug Administration (FDA) issued a proposed rule in January 2025 to require a nutrition information (Nutrition Info) interpretative front-of-package food labeling system that would indicate whether foods contain low (a positive attribute), medium, or high (a negative attribute) levels of saturated fat, sodium, and added sugar (ie, nutrients of concern).^10^ Few studies have evaluated these labels. While the FDA initially proposed requiring Nutrition Info labels, the agency also tested^11^ and could instead require “High In” front-of-package labels that signal when foods contain excessive levels of nutrients of concern (similar to labels used in Latin America^12^). The FDA could also consider other designs, such as spectrum labels that rate overall healthfulness from least to most healthy (similar to those used in Australia,^13^ France,^14^ and elsewhere). It remains unknown, however, whether these other labels outperform positive labels (the status quo for front-of-package labeling for many products in the US) or Nutrition Info labels.

To address these gaps, this randomized clinical trial evaluated whether Nutrition Info labels and other front-of-package labels lead to healthier purchases compared with positive labels and with one another. Additionally, because the FDA has emphasized that food labels should benefit people with lower nutrition literacy and social privilege,^10^ we evaluated whether label effects on healthfulness vary by nutrition literacy, household income, and educational attainment.

## Methods

### Participants

From October 31 through November 21, 2024, we recruited a sample of US adults through a survey research firm (CloudResearch Connect). Participants were eligible if they lived in the US, were 18 years or older, could read and speak English, and were their household’s primary shopper (ie, did >50% of their household’s grocery shopping). The Harvard Pilgrim Health Care Institutional Review Board approved this study, and all participants provided online informed consent. We preregistered the study prior to data collection, and the trial protocol is available in Supplement 1. We followed the Consolidated Standards of Reporting Trials (CONSORT) reporting guideline.

### Setting

The trial took place in a simulated online grocery store. The online store mirrored the look, feel, and functionalities of the e-commerce site used by a large US supermarket chain. The store included approximately 5300 products alongside their images, names, and prices as shown on the chain’s website in September 2023. Products were shown in the order in which they were displayed on the chain’s e-commerce site. Online stores such as this one are realistic, acceptable, and valid settings for nutrition research.^15,16^

### Procedures

Participants completed an online randomized clinical trial. After providing electronic informed consent, participants were randomized by performance management software (Qualtrics) using a simple allocation ratio to 1 of 6 trial arms representing different labeling systems: (1) positive, (2) Nutrition Info, (3) High In, (4) positive plus Nutrition Info, (5) positive plus High In, or (6) spectrum. We evaluated positive labels because they represent the status quo for voluntary food labels in the US and therefore offer a useful reference point against which to measure other labels. We evaluated Nutrition Info labels given that the FDA proposed these labels.^10^ We evaluated High In labels (which signal when foods exceed acceptable thresholds for nutrients of concern) and spectrum labels (which provide an overall rating of product healthfulness) because other countries have adopted these labels^12^ and because they may improve the healthfulness of food and beverage purchases.^17,18,19,20,21^ Finally, we evaluated the combination of positive plus Nutrition Info and positive plus High In labels because, if adopted, Nutrition Info and High In labels would likely appear alongside existing voluntary positive labels. Because positive labels are already commonly used, we considered the positive labels the reference group and the other 5 labeling systems the intervention labels.

**Figure 1.**
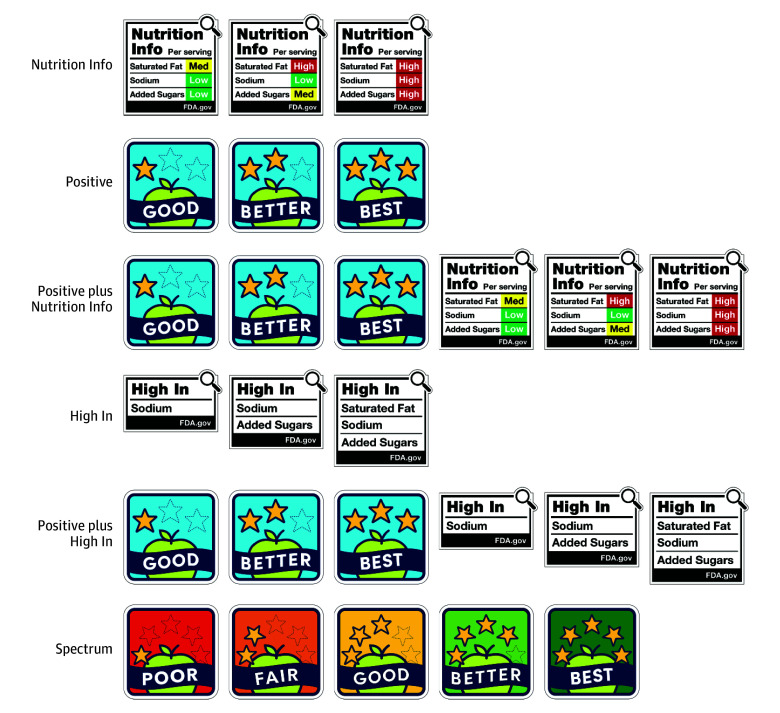
Examples of Labels Tested in the Trial The Nutrition Info and High In labels include the attribution FDA.gov but were developed by our team based on US Food and Drug Administration designs.

Figure 1 shows example labels and eTable 1 in Supplement 2 details criteria for assigning labels to products.^22^ The positive arm displayed Guiding Stars labels (which are among the most widely used positive labels in the US^23,24^) on products that met Guiding Stars’ criteria for earning at least 1 of 3 Guiding Stars.^25^ The Nutrition Info arm displayed Nutrition Info labels on all products. Like the FDA’s proposed design,^10^ the Nutrition Info labels we tested indicated whether the product contained low (<5% daily value [DV]), medium (5% to <20% DV), or high (≥20% DV) levels of saturated fat, sodium, and added sugar per serving.^10,11,26^ Our Nutrition Info labels mimicked designs the FDA tested^11^ but differed from their proposed label^10^ in 3 ways: we omitted percentage of DV information for simplicity, used traffic light color-coding to depict nutrient content levels, and included a magnifying glass icon. (eFigure in Supplement 2 shows an example of the FDA’s proposed label for comparison). The High In arm displayed High In labels when the product contained 20% or more of the DV per serving of saturated fat, sodium, or added sugar^26^; the labels mimicked designs that the FDA tested while developing their proposed rule.^11^ The positive plus Nutrition Info and positive plus High In arms displayed the Guiding Stars labels alongside the Nutrition Info and High In labels, respectively. The spectrum arm displayed modified Guiding Stars labels on all products with ratings ranging from least healthy (poor [1 star] with red labels) to most healthy (best [5 stars] with green labels), similar to other spectrum labels.^13,14^ The spectrum labels used the same color-coding as NutriScore,^14^ but unlike NutriScore, used words as ratings (eg, best, better) rather than letters (eg, A, B) and used a different underlying nutrient profile model.

Participants shopped in the online store. They were instructed to shop as they usually would for items in categories that are top contributors to intake of nutrients of concern in the US^27,28,29,30,31,32^: nonalcoholic beverages, boxed and frozen meals, breads and baked goods, cereals, snacks, and soups (eTable 2 in Supplement 2). Participants were assigned a budget of $35, similar to the median amount spent on these categories in our analyses of sales data from 2022 provided by the chain on which our store was designed. Participants could check out of the store if they were within 50% of the budget ($17.50-$52.50). Participants were informed that 1 in 50 would receive their selections and the remainder of their budget as a gift card; this procedure incentivized participants to select products they wished to receive. At the end of the study, participants were debriefed that those chosen to receive groceries would instead receive an electronic gift card for $35.

After completing the shopping task, participants responded to an online survey. They received an incentive worth US $5.50 from the survey research firm for completing the study.

### Measures

The primary outcome was the healthfulness of participants’ selections from the store, assessed using the United Kingdom’s Ofcom Nutrient Profiling Model (hereinafter referred to as Ofcom) scores.^33^ Under this model, foods and beverages are scored as healthier when they have lower levels of calories, sugar, saturated fat, and sodium density, higher levels of fiber and protein density, and higher fruit, vegetable, nut, and legume content. People who eat diets with healthier Ofcom scores are less likely to develop cardiovascular disease^34^ and metabolic syndrome.^35^ We calculated Ofcom scores for all products in the store, converting scores to a scale of 0 to 100 such that higher scores indicated healthier selections.^33^ We calculated mean Ofcom scores across all participants’ selections, weighted by the number of servings in each product so that scores better reflected expected consumption.

Secondary nutrition and selection outcomes included weighted mean Guiding Stars score of participants’ selections,^25^ the number of items selected that were high in 1 or more nutrients of concern, nutrient densities, nutrients per serving, total nutrient amounts, total number of items selected, and total spending in US dollars. Except for nutrients per serving, these outcomes were prespecified.

Other secondary outcomes were assessed using survey items. These included constructs that may explain how front-of-package labels exert their effects on behavior (eg, thinking about health),^36,37^ that the FDA measured in their research (eg, trustworthiness),^38^ and that may be of interest to policy-makers (ie, public support).

Finally, the survey assessed demographic characteristics (eg, race, ethnicity, educational attainment) and nutrition literacy (using 4 items from the Newest Vital Sign^39^). Race was self-reported by participants from the following options: American Indian or Alaska Native, Asian, Black or African American, Native Hawaiian or Other Pacific Islander, White, or more than 1 race or other (self-described by participants in a free-response box). Ethnicity was self-reported as Latino or Hispanic or non-Latino or non-Hispanic. Race and ethnicity data were collected to characterize the racial and ethnic composition of the sample and compare it to that of the US overall. eTable 3 in Supplement 2 shows survey measures.

### Statistical Analysis

First, we examined the effects of the front-of-package labels on the primary outcome of healthfulness using ordinary least squares regression. We regressed healthfulness on indicator variables for each trial arm, excluding the positive labels as the referent. We used the model to test whether the intervention labels led to healthier purchases than the positive labels, estimating average differential effects (ADEs; ie, differences in estimated means between groups) for each arm compared with the positive labels. We also tested whether the 5 intervention labels (excluding the positive labels) differed from one another in their effects on healthfulness.

Second, we examined whether the effects of the labels on healthfulness were moderated by nutrition literacy, household income, or educational attainment by adding the moderator and interactions between the moderator and the trial arms to the primary model and testing the joint significance of the interactions. Third, we examined the effects of the labels on the secondary outcomes using a similar approach as for the primary outcome, with ordinary least squares regression for continuous outcomes, logistic regression for binary outcomes, negative binomial regression for count outcomes (Poisson regression was preregistered but not used due to overdispersion), and mixed effects logistic regression for repeated-measures binary outcomes.

Statistical tests were 2 tailed. Analyses were conducted in Stata MP, version 19 (StataCorp LLC), from November 26, 2024, to August 27, 2025. Assuming a 2-tailed critical α value of .05, the target sample size of 5610 (935 per arm) yields 90% power to detect a difference in healthfulness between each intervention label and the positive labels of Cohen *d* = 0.15 or larger (based on prior studies^8,9,18^). We did not estimate statistical power for moderation analyses.

## Results

**Figure 2.**
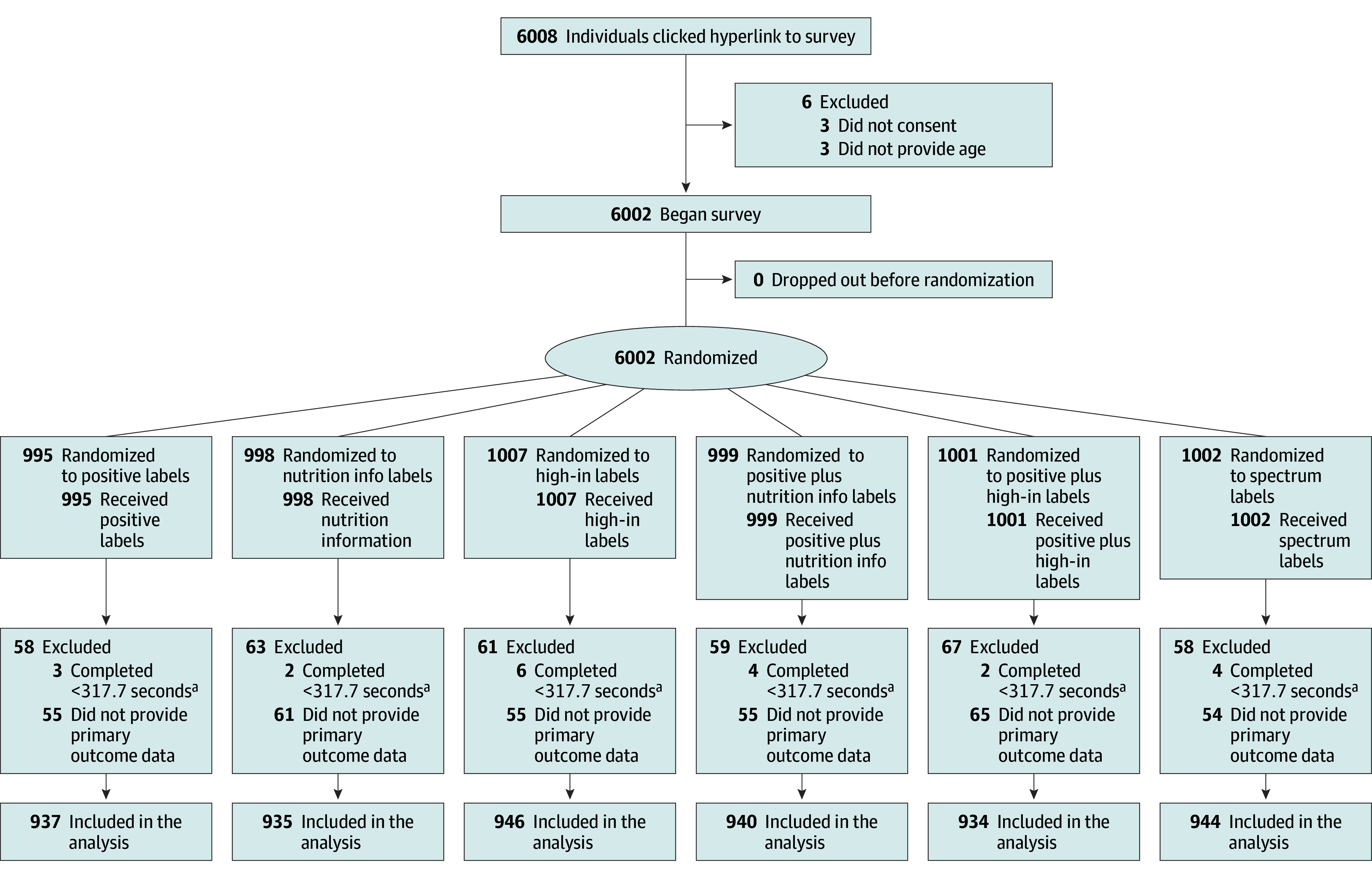
Study Flow Diagram ^a^Indicates one-third of the median completion time (15.88 [IQR, 11.50-22.54] minutes).

A total of 5636 participants (mean [SD] age, 40.3 [12.6] years) were included in the analyses (Figure 2). A total of 3400 participants (60%) identified as women, 2111 (38%) as men, and 118 (2%) as nonbinary or other, with 7 not responding. In terms of race, 35 participants (1%) identified as American Indian or Alaska Native; 337 (6%), Asian; 634 (11%), Black; 7 (0.1%), Native Hawaiian or Other Pacific Islander; 4305 (76%), White, and 310 (6%), multiracial or other, with 8 not responding. In terms of ethnicity, 580 participants (10%) identified as Latino or Hispanic (Table 1). Compared with the US overall, the sample included somewhat fewer older adults, men, people who identified as multiracial or other race, people with less than a college degree, and people with household income of $75 000 or greater (eTable 4 in Supplement 2).

**Table 1.** Participant Characteristics

Characteristic	Food and beverage label type, No. (%) of participants (N = 5636)^a^					
	Positive (n = 937)	Nutrition Info (n = 935)	High in (n = 946)	Positive plus Nutrition Info (n = 940)	Positive plus High In (n = 934)	Spectrum (n = 944)
Age, y						
18-29	211 (23)	200 (21)	183 (19)	191 (20)	188 (20)	201 (21)
30-44	426 (45)	456 (49)	416 (44)	438 (47)	454 (49)	449 (48)
45-59	213 (23)	199 (21)	240 (25)	222 (24)	195 (21)	211 (22)
≥60	87 (9)	80 (9)	107 (11)	89 (9)	97 (10)	83 (9)
Gender						
Men	348 (37)	365 (39)	350 (37)	330 (35)	351 (38)	367 (39)
Women	574 (61)	539 (58)	582 (62)	596 (63)	563 (60)	546 (58)
Nonbinary or other gender^b^	14 (1)	29 (3)	13 (1)	14 (1)	17 (2)	31 (3)
Latino or Hispanic ethnicity	97 (10)	91 (10)	92 (10)	97 (10)	99 (11)	104 (11)
Race						
American Indian or Alaska Native	5 (1)	4 (0.4)	6 (1)	9 (1)	8 (1)	3 (0.3)
Asian	53 (6)	56 (6)	66 (7)	49 (5)	52 (6)	61 (6)
Black or African American	108 (12)	87 (9)	92 (10)	114 (12)	121 (13)	112 (12)
Native Hawaiian or Pacific Islander	0	1 (0.1)	1 (0.1)	1 (0.1)	1 (0.1)	3 (0.3)
White	732 (78)	736 (79)	729 (77)	703 (75)	703 (76)	702 (74)
Other or multiracial^c^	38 (4)	48 (5)	51 (5)	64 (7)	46 (5)	63 (7)
Educational attainment						
High school diploma or less	114 (12)	103 (11)	99 (10)	118 (13)	103 (11)	106 (11)
Some college	176 (19)	198 (21)	181 (19)	185 (20)	231 (25)	224 (24)
College graduate or associate degree	492 (53)	461 (49)	502 (53)	493 (52)	469 (50)	453 (48)
Graduate degree	154 (16)	171 (18)	163 (17)	144 (15)	128 (14)	161 (17)
Household size						
1-2	472 (50)	510 (55)	513 (54)	489 (52)	468 (50)	512 (54)
3-4	380 (41)	329 (35)	350 (37)	358 (38)	365 (39)	321 (34)
≥5	83 (9)	91 (10)	80 (8)	92 (10)	98 (11)	108 (11)
Annual household income, $						
0-24 999	108 (12)	129 (14)	129 (14)	120 (13)	123 (13)	116 (12)
25 000-49 999	205 (22)	202 (22)	193 (21)	200 (21)	218 (23)	188 (20)
50 000-74 999	202 (22)	180 (19)	216 (23)	204 (22)	191 (20)	201 (21)
≥75 000	420 (45)	418 (45)	403 (43)	415 (44)	399 (43)	436 (46)
Nutrition literacy score, mean (SD)^d^	3.3 (1.0)	3.3 (1.0)	3.3 (1.0)	3.3 (1.0)	3.2 (1.0)	3.3 (1.0)

^a^ Percentages may not sum to 100 due to rounding and missing data (≤20 [0.4%]).

^b^ Includes any identity participants self-described in a free-response box.

^c^ Includes any identity participants self-described in a free-response box.

^d^ Based on responses to the first 4 questions from the Newest Vital Sign^39^ (eTable 3 in Supplement 2 provides survey measures).

**Figure 3.**
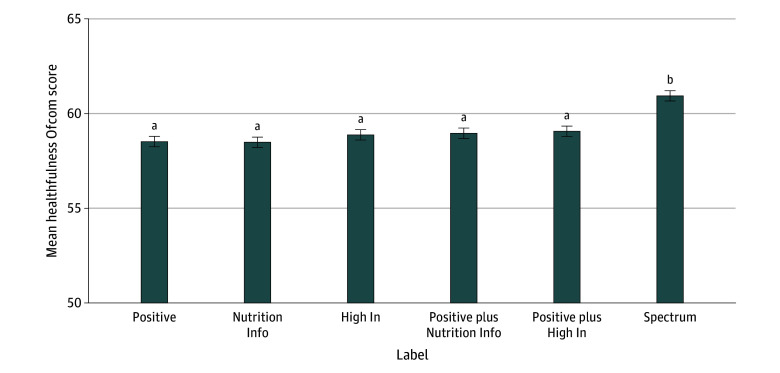
Healthfulness of Food and Beverage Purchases by Front-of-Package Labeling System Includes 5636 US adults. Ofcom Nutrient Profiling Model scores range from 0 to 100, with higher scores indicating healthier choices. ^a^Means are not different from one another (*P* ≥ .14). ^b^*P* < .001 compared with all other means.

Participants exposed to the positive labels selected groceries with overall healthfulness (Ofcom score) of 58.5 of 100 (Figure 3 and eTable 5 in Supplement 2). The Nutrition Info labels did not improve healthfulness compared with the positive labels (ADE, −0.04 [95% CI, −0.80 to 0.72]; *P* = .92) (Table 2). Similarly, the High In (ADE, 0.36 [95% CI, −0.40 to 1.11]; *P* = .36), positive plus Nutrition Info (ADE, 0.44 [95% CI, −0.32 to 1.20]; *P* = .26), and positive plus High In (ADE, = 0.54 [95% CI, −0.22 to 1.30]; *P* = .16) labels did not improve healthfulness compared with the positive labels. By contrast, the spectrum labels improved healthfulness by 2.42 (95% CI, 1.66-3.17) points compared with the positive labels (*P* < .001). The effects of the labels on overall healthfulness were not moderated by nutrition literacy (*P* for interaction = .82), household income (*P* for interaction = .29), or educational attainment (*P* for interaction = .64) (eTable 6 in Supplement 2).

**Table 2.** Food and Beverage Selections by Front-of-Package Labeling System^a^

Outcome	Differences vs positive labels, ADE (95% CI)				
	Nutrition Info	High in	Positive plus Nutrition Info	Positive plus High In	Spectrum
Increase					
Healthfulness, Ofcom score^b^	−0.04 (−0.80 to 0.72)	0.36 (−0.40 to 1.11)	0.44 (−0.32 to 1.20)	0.54 (−0.22 to 1.30)	2.42 (1.66 to 3.17)^c^
Guiding Stars score^d^	−0.41 (−0.65 to −0.17)^c^	0.04 (−0.21 to 0.28)	−0.18 (−0.42 to 0.06)	0.21 (−0.04 to 0.45)	0.77 (0.53 to 1.01)^c^
Fiber density, g/100 g	−0.08 (−0.27 to 0.11)	0.06 (−0.13 to 0.25)	−0.16 (−0.35 to 0.03)	−0.07 (−0.26 to 0.12)	0.24 (0.05 to 0.43)^c^
Protein density, g/100 g	−0.12 (−0.41 to 0.17)	0.22 (−0.07 to 0.51)	−0.17 (−0.46 to 0.12)	0.002 (−0.29 to 0.29)	0.29 (−0.001 to 0.58)
Decrease					
No. of items high in ≥1 nutrient of concern	0.11 (−0.16 to 0.38)	−0.39 (−0.65 to −0.14)^c^	−0.07 (−0.33 to 0.19)	−0.47 (−0.72 to −0.22)^c^	−0.43 (−0.69 to −0.18)
Calorie density, kcal/100 g	−5.28 (−14.07 to 3.51)	−6.00 (−14.77 to 2.77)	−10.62 (−19.40 to −1.84)^c^	−7.87 (−16.66 to 0.93)	−15.02 (−23.79 to −6.25)^c^
Sugar density, g/100 g	0.17 (−0.51 to 0.84)	−0.43 (−1.10 to 0.24)	0.04 (−0.64 to 0.71)	−0.38 (−1.06 to 0.29)	−0.49 (−1.16 to 0.18)
Saturated fat density, g/100 g	−0.11 (−0.34 to 0.11)	0.07 (−0.16 to 0.29)	−0.12 (−0.35 to 0.10)	−0.11 (−0.33 to 0.12)	−0.41 (−0.64 to −0.19)^c^
Sodium density, mg/100 g	10.11 (−11.45 to 31.66)	12.96 (−8.53 to 34.45)	−12.19 (−33.71 to 9.34)	−9.82 (−31.38 to 11.74)	−35.96 (−57.46 to −14.46)^c^
Neutral					
No. of items selected	0.19 (−0.12 to 0.50)	−0.04 (−0.35 to 0.26)	−0.04 (−0.34 to 0.27)	−0.05 (−0.35 to 0.26)	−0.14 (−0.44 to 0.16)
Spending, US $	0.01 (−0.43 to 0.45)	−0.15 (−0.58 to 0.29)	−0.14 (−0.58 to 0.29)	−0.12 (−0.56 to 0.31)	−0.43 (−0.87 to 0.002)

Abbreviation: ADE, average differential effect.

^a^ Includes 5636 US adults.

^b^ Scores range from 0 to 100, with higher scores indicating healthier choices.

^c^ Statistically significant at *P* < .05.

^d^ Scores range from −22 to 7, with higher scores indicating healthier foods and beverages.

When comparing the intervention labels with one another, the spectrum labels were more effective at improving healthfulness than the Nutrition Info, High In, positive plus Nutrition Info, and positive plus High In labels (ADE range, −1.87 [95% CI, −2.63 to −1.11] to −2.45 [95% CI, −3.21 to −1.69]; *P* < .001) (Figure 3 and eTable 7 in Supplement 2). There were no other differences in overall healthfulness between the labels.

In addition to leading to healthier selections as assessed by Ofcom scores, the spectrum labels also outperformed the positive labels for most secondary selection outcomes, including leading to selections with healthier Guiding Stars scores, higher fiber density, more fiber per serving, more total fiber, more protein per serving, fewer items that were high in 1 or more nutrients of concern, lower calorie density, less total sugar, lower saturated fat density, less saturated fat per serving, lower sodium density, and less total sodium (Table 2 and eTable 8 in Supplement 2). For example, the spectrum labels led to selections with 1189.38 mg (95% CI, −1803.85 to −574.92 mg) less total sodium compared with the positive labels (*P* < .001), an approximate 10% reduction compared with total sodium levels selected in the positive labels arm (approximately 11 865 mg) (eTable 5 in Supplement 2).

By contrast, the other intervention labels—including the Nutrition Info labels—generally did not improve these outcomes compared with the positive labels, and sometimes worsened them (eg, the Nutrition Info labels led to selections with more sodium per serving). There were exceptions to this pattern. First, the positive plus Nutrition Info labels led to selections with lower calorie density compared with the positive labels. Second, the High In and positive plus High In labels led to selections with fewer items high in 1 or more nutrients of concern, and the High In labels led to selections with less sugar per serving and less total sugar. None of the intervention labels led to differences in the total number of items selected or total spending in the shopping task compared with the positive labels (Table 2 and eTable 8 in Supplement 2).

The intervention labels generally outperformed the positive labels on psychological outcomes. For example, each of the intervention labels generally led to higher likelihood of noticing and using the labels, more thinking about health, more negative emotions, and stronger perceptions that the labels were helpful, trustworthy, and understandable (eTable 9 in Supplement 2).

## Discussion

In this randomized clinical trial in a large sample of US adults, spectrum food labels that rated all foods from least to most healthy led participants to select healthier foods compared with the status quo of displaying only positive labels on healthier foods. Spectrum labels also led to healthier selections compared with other designs for new labels, including a Nutrition Info label similar to that proposed by the FDA. Although the benefits of the spectrum labels may appear modest (improvements of approximately 2-3 points on the Ofcom scale), prior epidemiological research finds that each 2-point increase in diet quality on the 0- to 100-point Ofcom scale is associated with a 14% lower risk of developing cardiovascular disease.^34^ Moreover, the effects of the food labels on healthfulness did not vary by nutrition literacy, household income, or educational attainment. Together, these results suggest that adopting spectrum labels could lead to population health benefits without exacerbating health disparities.

The spectrum labels led to overall healthier selections compared with all other labeling systems we tested, similar to prior studies.^18^ The spectrum labels may have outperformed the positive and High In labels because spectrum labels were applied to all products (rather than only to healthier or less healthy products), so consumers did not need to speculate about the healthfulness of products without labels. The Nutrition Info labels were also displayed on all products; the spectrum labels may have outperformed those labels because they provided a single summary interpretation of product healthfulness (eg, fair, good), while the Nutrition Info labels required consumers to integrate potentially conflicting information about 3 different nutrients. It is less clear why the spectrum labels outperformed the positive plus High In labels, given that both conditions included labels conveying both positive and negative attributes. One possibility is that the spectrum labels, which summarize foods’ healthfulness on a single scale on all products, were simpler to understand than the combination of positive and High In labels, which use different scales and are not both present on all products. However, we cannot rule out that other aspects of label design drove this difference.

The labels we studied differed not only in their visual design, but also in the underlying nutritional model defining how labels are applied. The Nutrition Info and High In labels are applied based on levels of nutrients per serving, while the positive and spectrum labels are applied based on nutrient and ingredient densities. Our primary outcome (Ofcom scores) reflected healthfulness based on nutrient densities, potentially favoring the spectrum labels over the Nutrition Info and High In labels. However, even when examining outcomes based on nutrients per serving, the spectrum labels led to the best outcomes overall, followed by the High In labels. By contrast, the Nutrition Info labels did not improve any healthfulness measures based on either nutrient densities or nutrients per serving. These results suggest that spectrum labels, and to a lesser extent High In labels, are more promising for promoting healthier purchases than Nutrition Info labels, regardless of how healthfulness is defined.

In our study, High In labels did not lead to overall healthier selections than positive labels but did lead to selections with fewer items high in 1 or more nutrients of concern, less sugar per serving, and less total sugar. Robust evidence indicates that High In labels as designed by many Latin American countries lead to meaningful improvements in the overall healthfulness of food and beverage purchases.^9,17^ The more limited benefits from the High In labels in our study may reflect that our High In labels (which were modeled after the FDA’s designs^11^) were depicted on a single white square-shaped label, while Latin American High In labels are typically depicted on multiple black octagon-shaped labels. If the FDA pursues High In labels, designs like those used in Latin American labels may be more effective.^40^

### Strengths and Limitations

Strengths of this study include the large sample, randomized design, and use of a realistic online grocery store. Limitations include that participants had a single exposure to the labels in the context of an online store; effects after repeated exposures and in brick-and-mortar settings remain unknown. Second, we recruited a convenience sample, although randomized clinical trials conducted with convenience samples generally replicate in representative samples.^41,42^ Third, although we incentivized participants to select groceries they wished to receive, participants did not pay for or receive their selections. Fourth, the different labeling systems sometimes varied more than 1 characteristic simultaneously, making it more challenging to untangle why one system outperformed another. For example, the spectrum labels used a different color scheme for good, better, and best labels than the positive labels and also rated all products (not just healthier ones). Future research should systematically vary all elements of label design to isolate the aspects driving differences in effects. Fifth, we developed a new spectrum label to mimic the positive labels, rather than using an established labeling system like NutriScore; future research could compare our spectrum label to existing systems.

## Conclusions

In this randomized clinical trial, spectrum food labels that rated foods from least to most healthy led participants to select healthier foods compared with positive, Nutrition Info, and High In labels. These findings suggest that spectrum labels may be more promising than both existing positive labels and the FDA’s proposed Nutrition Info labels for promoting healthier food and beverage purchases.

## Notes

Supplement 1.
Trial Protocol

Supplement 2. **eFigure.** Example of FDA’s Proposed Nutrition Info Label **eTable 1.** Criteria for Assigning Labels to Each Product **eTable 2.** Number and Types of Products Displayed in the Departments and Categories in the Naturalistic Online Store **eTable 3.** Survey Measures Used in the Trial **eTable 4.** Comparison of Characteristics of the Study Sample (5636 US Adults) to National Estimates **eTable 5.** Healthfulness and Nutrient Content of and Spending on Food and Beverage Selections, by Front-of-Package Labeling System (5636 US Adults) **eTable 6.** Impact of Front-of-Package Labeling Systems on Healthfulness of Food and Beverage Purchases, by Nutrition Literacy, Household Income, and Educational Attainment (5636 US Adults) **eTable 7.** Comparison of Effects of Novel Front-of-Package Labeling Systems on Healthfulness of Food and Beverage Purchases (5636 US Adults) **eTable 8.** Impact of Front-of-Package Labeling Systems on Secondary Selection Outcomes (5636 US Adults) **eTable 9.** Impact of Front-of-Package Labeling Systems on Consumer Understanding, Label Reactions and Perceptions, and Public Support (5636 US Adults)
eReferences

Supplement 3.
Data Sharing Statement
