# Supplementary material for: Nutrition Info and Other Front-of-Package Labels and Simulated Food and Beverage Purchases: A Randomized Clinical Trial
Source: JAMA Netw Open. 2025 Oct 17;8(10):e2537389. doi: 10.1001/jamanetworkopen.2025.37389 (PMC12534843; doi:10.1001/jamanetworkopen.2025.37389)
Supplement: Supplement 1. — Trial Protocol [file jamanetwopen-e2537389-s001.pdf]

Effect of “Nutrition Info” and other front-of-package labels on simulated food and beverage purchases: A randomized clinical trial

Notes

Per journal instructions, this document contains the trial protocol and analysis plan as it was submitted to the Harvard Pilgrim Health Care Institute Institutional Review Board. We additionally pre-registered the analysis plan through ClinicalTrials.gov (NCT#06516627). Deviations from this plan are detailed in the main text.

Label to Table: New Insights into Supermarket Food Labeling

IRB Protocol

<u>Aim 3a: Online randomized trial</u>

3.3.6.2. Design/Intervention. We will recruit 5,610 adults to participate in a randomized-controlled trial during which they will shop in a naturalistic online grocery store. Participants will be randomized to one of six labeling arms: 1) current 2021 Guiding Stars endorsement logos (1-3 stars); 2) Expanded Guiding Stars system with positive and negative ratings (unhealthy to healthy; 1-5 stars); 3) High In labels (labels placed on foods high in saturated fat, sodium, or added sugars); and 4) Current Guiding Stars + High In labels (combination of conditions 1 and 3); 5) Traffic light; and 6) Traffic light + Current Guiding Stars (**Figure 3**). The Guiding Stars team will help develop final design and language for the expanded system based on their prior market research.

Figure 3: Conditions for experiment

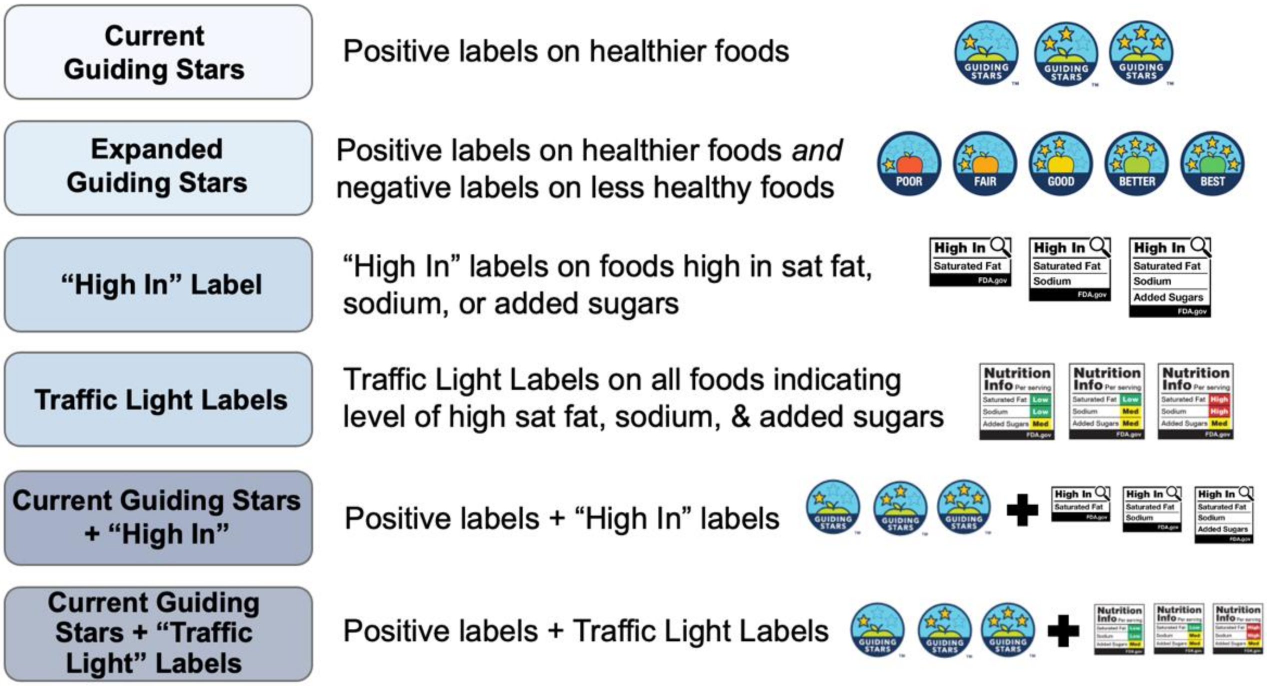

3.3.6.3 Setting. We will develop a naturalistic online supermarket, using their online research shopping platform[110–113] We will develop a store to emulate the appearance and function (e.g., browsing, search) of Hannaford.com, to match the products for which we have sales and nutrition data. To populate the store with items to sell, we will follow standardized steps:[114] compiling a dataset of items sold by Hannaford.com using web scraping; organizing products into departments, aisles, and shelves; compiling nutrition information for products; and completing quality control checks. Products will have real-world prices (from Hannaford.com) held constant across conditions. Based on our experience analyzing product-level data from similar retailers,[11,67–69] we anticipate the store will include hundreds of products in each food category of interest described below. Each page will display up to 60 products (each with an image, price and randomly assigned label) sorted by "shelf rank" (popularity) as collected from Hannaford.com.

3.3.6.4 Participants and Procedures. We will recruit 5,610 adults through Cloud Research, a survey research firm with whom we have worked previously. Eligibility criteria will be: 1) ≥18 years old; 2) can read and speak English; and 3) primary shopper (do >50% of households' grocery shopping). To ensure adequate sample sizes for assessing potential disparities in effect, we aim to recruit a sample that is ~25% Hispanic and ~25% non-Hispanic Black. Cloud Research will use quota-based recruitment to ensure that the sample approximately reflects the age, gender, education and income distributions of the US population of primary shoppers.

After providing informed consent, participants will be randomized to one of the six labeling arms and enter the online grocery store platform. In all arms, products will be displayed with the assigned labels shown below the item image as on Hannaford.com. Participants will be asked to imagine they are shopping for items for their household in the following categories: 1) bread, 2) breakfast cereal, 3) beverages, 4) sweet and salty snacks, 5) soup, and 6) cold convenience foods or meal kits. We selected these categories because they are top contributors to calories,[119] added sugar,[120] saturated fat,[119] or sodium[121] intake and because they offer a range of less to more healthy foods, providing consumers an opportunity to substitute toward healthier products. To encourage more realistic shopping behaviors, participants will be given the option to skip all products in a category. Average spending on these categories in 2021 was $30.82/transaction in our sales data. Thus, the suggested budget for the shopping task will be $30, and participants will only be allowed to exit the store once they have spent between 50% ($15.00) and 150% ($45.00) of this budget. Participants will indicate their selections by adding them to their shopping cart. No payment will be required. All will receive a participation incentive determined by Cloud Research.

To encourage responses similar to real-world shopping, participants will be told in advance that 1 in 50 individuals will be chosen via lottery to have their selections shipped to them. This procedure enhances external validity because participants are incentivized to shop as if in a real store. After finishing the shopping task, participants will answer survey items (**see attached survey measures**). At the end of the study, participants who won the lottery will be provided with a gift card equivalent to the amount they spent in the shopping task instead of having the groceries delivered. This will overcome the logistical challenges of delivering groceries while still ensuring participants' selections are items they wish to receive. Our prior studies have found that this procedure is acceptable to participants. Only 2-3% of participants asked to have their data removed from these studies after learning of this procedure. Moreover, naturalistic online grocery platforms like the one proposed for this study have high external validity: shopping behaviors in these settings are highly comparable to those in actual stores.[111,114,130,131] For example, one study found that spending on eight food categories in a naturalistic online supermarket was highly correlated with average amounts spent in national supermarket data ($r=0.85$, $p<0.05$).[132]

3.3.6.5 Outcomes and Statistical Analyses. The primary outcome will be overall healthfulness of purchases, measured with the UK Ofcom Nutrient Profiling Model (UK NPM).[74,133] We will calculate the average NPM score for all products selected. Our primary hypothesis is that the

Expanded Positive and Negative, High In, Traffic Light, Current Guiding Stars + High In, and Traffic Light + Current Guiding Stars labeling systems will lead to healthier purchases than the Current 2021 Guiding Stars label. We will use repeated measures ANOVA or mixed effects linear regression to test hypotheses, using appropriate contrasts to compare purchase healthfulness across randomized groups. To assess the potential for labels to affect disparities, secondary analyses will estimate treatment effects by race, ethnicity, income, education, and nutrition literacy. The target total sample size of 5,610 (935 per arm) would yield 90% power to detect an effect of each front-of-package labeling systems vs. the positive labeling system of d=.15 (based on prior studies of similar labeling systems) or larger assuming a two-tailed critical alpha of .05.
