# Supplementary material for: Nutrition Info and Other Front-of-Package Labels and Simulated Food and Beverage Purchases: A Randomized Clinical Trial
Source: JAMA Netw Open. 2025 Oct 17;8(10):e2537389. doi: 10.1001/jamanetworkopen.2025.37389 (PMC12534843; doi:10.1001/jamanetworkopen.2025.37389)
Supplement: Supplement 2. — eFigure. Example of FDA’s Proposed Nutrition Info Label eTable 1. Criteria for Assigning Labels to Each Product eTable 2. Number and Types of Products Displayed in the Departments and Categories in the Naturalistic Online Store eTable 3. Survey Measures Used in the Trial eTable 4. Comparison of Characteristics of the Study Sample (5636 US Adults) to National Estimates eTable 5. Healthfulness and Nutrient Content of and Spending on Food and Beverage Selections, by Front-of-Package Labeling System (5636 US Adults) eTable 6. Impact of Front-of-Package Labeling Systems on Healthfulness of Food and Beverage Purchases, by Nutrition Literacy, Household Income, and Educational Attainment (5636 US Adults) eTable 7. Comparison of Effects of Novel Front-of-Package Labeling Systems on Healthfulness of Food and Beverage Purchases (5636 US Adults) eTable 8. Impact of Front-of-Package Labeling Systems on Secondary Selection Outcomes (5636 US Adults) eTable 9. Impact of Front-of-Package Labeling Systems on Consumer Understanding, Label Reactions and Perceptions, and Public Support (5636 US Adults) eReferences [file jamanetwopen-e2537389-s002.pdf]

Supplementary Online Content

Grummon AH, O'Sullivan K, Petimar J, et al. Nutrition Info and other front-of-package labels and simulated food and beverage purchases: a randomized clinical trial. *JAMA Netw Open.* 2025;8(10):e253789. doi:10.1001/jamanetworkopen.2025.37389

eFigure. Example of FDA's Proposed Nutrition Info Label
eTable 1. Criteria for Assigning Labels to Each Product
eTable 2. Number and Types of Products Displayed in the Departments and Categories in the Naturalistic Online Store
eTable 3. Survey Measures Used in the Trial
eTable 4. Comparison of Characteristics of the Study Sample (5636 US Adults) to National Estimates
eTable 5. Healthfulness and Nutrient Content of and Spending on Food and Beverage Selections, by Front-of-Package Labeling System (5636 US Adults)
eTable 6. Impact of Front-of-Package Labeling Systems on Healthfulness of Food and Beverage Purchases, by Nutrition Literacy, Household Income, and Educational Attainment (5636 US Adults)
eTable 7. Comparison of Effects of Novel Front-of-Package Labeling Systems on Healthfulness of Food and Beverage Purchases (5636 US Adults)
eTable 8. Impact of Front-of-Package Labeling Systems on Secondary Selection Outcomes (5636 US Adults)
eTable 9. Impact of Front-of-Package Labeling Systems on Consumer Understanding, Label Reactions and Perceptions, and Public Support (5636 US Adults)
eReferences

This supplementary material has been provided by the authors to give readers additional information about their work.

© 2025 Grummon AH et al. *JAMA Network Open*.

eFigure. Example of FDA's Proposed Nutrition Info Label

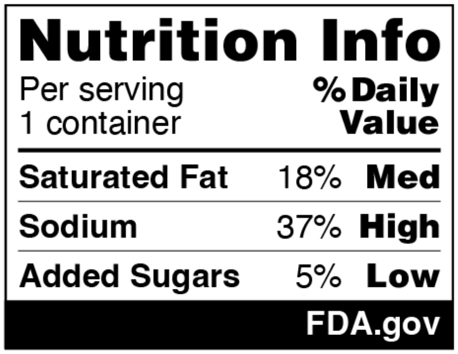

© 2025 Grummon AH et al. *JAMA Network Open*.

eTable 1. Criteria for Assigning Labels to Each Product

Labeling system	Criteria for assigning labels
Positive	Products that met Guiding Stars' criteria for earning 1 or more Guiding Star displayed labels indicating their healthfulness using star ratings: 1 star ("Good"), 2 stars ("Better"), or 3 stars ("Best"). The Guiding Stars rating system uses a patented algorithm to rate foods based on their nutrients and ingredients per 100-calorie serving of the food.[3] Foods earn points for healthier nutrients and ingredients including vitamins, minerals, fiber, whole grains, and omega-3s, and lose points for unhealthy nutrients and ingredients including saturated fat, trans fat, added sodium, added sugars, and certain additives. Scores for products offered in the store ranged from -22 to 7. Guiding Stars evaluates all products and provides each a rating, but only those with a positive rating earn 1, 2, or 3 Guiding Stars. Products that did not meet Guiding Stars' criteria for earning stars did not display any new front-of-package label. We obtained information on Guiding Stars ratings from Guiding Stars, LLC.
Nutrition Info	All products displayed a "Nutrition Info" label showing whether the amount of saturated fat, sodium, and added sugars in 1 serving of the product was low (<5% DV), medium (≥5 to <20% DV), or high (≥20% DV).[1] "Low" was shown in green, "Medium" in yellow, and "High" in red. We used DVs of 20g for saturated fat, 2,300mg for sodium, and 50g for added sugars per FDA definitions.[2]
High In	Products that contained high levels (i.e., ≥20% DV per serving) of saturated fat, sodium, or added sugars displayed a label indicating which of these nutrients the product was high in. Products that did not contain high levels of saturated fat, sodium, or added sugar did not display any new front-of-package label. We used DVs of 20g for saturated fat, 2,300mg for sodium, and 50g for added sugars per FDA definitions.[2]
Spectrum	All products displayed an expanded Guiding Stars label that showed an overall rating indicating their healthfulness using star ratings: 1 star ("Poor"), 2 stars ("Fair"), 3 stars ("Good"), 4 stars ("Better"), or 5 stars ("Best"). The Guiding Stars rating system is described above. Products that displayed 1-, 2-, or 3-star labels in the Positive Labels (Current Guiding Stars) system (see above) displayed 3-, 4-, or 5-star labels, respectively, in the Spectrum Labels (Expanded Guiding Stars) system. We divided the remaining products that did not earn stars in the Positive Labels system in half based in their underlying Guiding Stars scores; the bottom half of these products displayed 1-star labels and the top half of these products displayed 2-star labels.

© 2025 Grummon AH et al. *JAMA Network Open*.

eTable 2. Number and Types of Products Displayed in the Departments and Categories in the Naturalistic Online Store

Food group	Department	Category	Description of included products	Number of products displayed
Beverages	Beverages	Coffee, tea & cocoa	Pre-packaged coffees and teas, tea bags tea, coffee pods, hot chocolate mix, coffee creamers, flavored syrups, cider, instant coffee	191
	Beverages	Juice	100% fruit and vegetable juice, juice blends	202
	Beverages	Powdered drinks	Powdered drink mixes, diet powdered drink mixes, water enhancers	45
	Beverages	Soda	Sodas, diet sodas	201
	Beverages	Sports Drinks	Sports drinks, diet sports drinks, energy drinks, diet energy drinks	104
	Beverages	Water	Plain bottled water, seltzer water, flavored water, coconut water	62
Boxed and frozen meals	Grocery	Convenience meals	Dinner kits and sides, macaroni and cheese, ready-to-heat meals, taco seasoning, taco shell kits and tortillas	257
	Frozen	Frozen dinners & entrees	Frozen pasta, frozen pot pies, frozen macaroni and cheese, frozen chicken nuggets, frozen patties, frozen meals	398
	Frozen	Frozen meat, chicken & seafood	Frozen chicken, frozen turkey, frozen fish sticks, frozen breaded fish	154
	Frozen	Frozen pizza, sandwiches & snacks	Frozen pizza, frozen burritos, frozen sandwiches	262
	Meat	Frozen Meat & Chicken	Frozen burgers, frozen chicken breast, frozen sausages	45
	Frozen	Frozen potatoes, fries & onion rings	Frozen tater tots, frozen French fries, frozen hashbrowns	46
Breads and baked goods	Grocery	Packaged bread, muffins & pastries	Bagels, bread, buns, English muffins, hamburger and hot dog buns, rolls, snack cakes and pastries, tortillas, wraps	336
	Frozen	Frozen breads & doughs	Frozen bagels, frozen bread, frozen biscuits, frozen dough, frozen garlic bread, frozen rolls	46
Cereals	Grocery	Breakfast & cereal	Breakfast bars, breakfast drinks, cold cereal, granola, oatmeal and hot cereal, pancakes, toaster pastries, waffles, syrup	789
Snacks	Grocery	Snacks, chips, crackers & nuts	Crackers, dips and salsas, fruit snacks, meat snacks and jerky, nuts, seeds, popcorn, potato chips, pretzels, rice cakes, snack mixes, tortilla chips	1511
	Grocery	Applesauce & fruit	Applesauce, canned fruit, fruit cups, fruit pouches, dried fruit (raisins, cranberries, apricots, mango, etc.) (note, does not include fresh fruit)	238
Soups	Grocery	Soups	Broth, bouillon, condensed soup, ramen, ready-to-serve soup, soup mixes	431

© 2025 Grummon AH et al. *JAMA Network Open*.

eTable 3. Survey Measures Used in the Trial

Construct	Item<br>[programming notes]	Response scale<br>[programming notes]
	**Eligibility**	
Age	How old are you?	**[Force response]**<br>[free response, #, restricted whole numbers to 0-115]<br><br>**[if <18, skip to termination screen]**
	**Introduction to Shopping Task**	
Prompt	Next we will explain the online supermarket and shopping task.	
Prompt	You will now view an online grocery store and complete a shopping task. Then, you will answer survey questions.<br><br>The online grocery store looks like a typical store, **but only offers the following types of foods**:<br>• Beverages<br>• Breads<br>• Cereals<br>• Soups<br>• Boxed and frozen meals<br>• Snacks<br><br>Enter the online store and select the items you would like to purchase among the types of foods offered in this store. You should shop as you normally would for these types of foods. For example, if you usually buy bread but not soup, you should select your preferred bread from the store, but you don't need to select a soup.<br><br>**Please spend about $35 or less.** You will not be asked to spend your own money. However, you should make your selections carefully because **we will choose 1 in 50 participants to receive the groceries they selected, plus any money left over.** In total, about 100 participants will receive the groceries they selected, plus any money left over in their budget. This means you should select products you actually want.	

© 2025 Grummon AH et al. *JAMA Network Open*.

Construct	Item<br>[programming notes]	Response scale<br>[programming notes]
Validate understanding of shopping task	We care about the quality of our data. For us to get the most accurate information, it is important that you understand these instructions.<br><br>Please select the answer option that is correct based on the information you have read above:<br><br>**[page break]**	**[Randomize order of responses]**<br><br>1=I have to pay for the items I select with my own money<br>2=There is a chance I will be chosen to receive the groceries I selected<br>3=No participants in this study will receive the groceries they selected<br><br>**[validate response such that participants cannot continue until they select response option 2. Use custom validation text that says, "Your response was incorrect. Please read the instructions carefully and try again."]**
Additional shopping task instructions	You will now enter the online grocery store. The store can take up to 30 seconds to load on a fast internet connection and a bit longer on a slower internet connection. Thank you for your patience.<br><br>Once you enter the store, please shop as you normally would. Remember that the store only offers certain types of foods (e.g., cereal, bread). Your budget is $35. If you have questions while you shop, click the "help" button at the top right side of the online store.<br><br>Click the next button to be re-directed to the store.	
Randomization	**[Participants are randomized via Qualtrics to 1 of the 6 labeling conditions]**	
	**Shopping Task**	
Programming notes	**[participants complete shopping task in the naturalistic online store, then return to the Qualtrics survey to complete the rest of the survey questions]**	
	**Introduction to Rest of Survey**	
Prompt	**Thank you for completing the shopping task! You will now answer some questions.**<br><br>[page break]	
	**Consumer Understanding - Introduction**	
Prompt	The next questions are about different products. You may or may not have seen these products during the shopping task.	
Programming notes	**[display labels from participants randomly assigned trial arm]**<br>**[randomize order of all questions]**	
Identification of healthier product – beverages	Click on the product you think is **healthier,** or click "they're the same" if you think the two products are equally healthy.<br><br>**[page break]**	[randomize order of 1 and 2, display 3 last]<br>1=[Display image of Ocean Spray 100% Cranberry juice]<br>2=[Display image of Ocean Spray Cranberry Juice Cocktail]<br>3=They're the same

© 2025 Grummon AH et al. *JAMA Network Open*.

Construct	Item<br>[programming notes]	Response scale<br>[programming notes]
Identification of healthier product – breads	Click on the product you think is **healthier,** or click “they’re the same” if you think the two products are equally healthy.<br><br>**[page break]**	[randomize order of 1 and 2, display 3 last]<br>1=[Display image of Mission Extra Thin Corn Tortillas]<br>2=[Display image of Mission Flour Burrito Tortillas]<br>3=They’re the same
Identification of healthier product – cereals	Click on the product you think is **healthier,** or click “they’re the same” if you think the two products are equally healthy.<br><br>**[page break]**	[randomize order of 1 and 2, display 3 last]<br>1=[Display image of Post Selects Great Grains Crunchy Pecans]<br>2=[Display image of General Mills Chocolate Chex Gluten Free Cereal]<br>3=They’re the same
Identification of healthier product – soups	Click on the product you think is **healthier,** or click “they’re the same” if you think the two products are equally healthy.<br><br>**[page break]**	[randomize order of 1 and 2, display 3 last]<br>1=[Display image of Campbell’s Healthy Request Chunk Sirloin Burger Soup]<br>2=[Display image of Dinty Moore Beef Stew]<br>3=They’re the same
Identification of healthier product – snacks	Click on the product you think is **healthier,** or click “they’re the same” if you think the two products are equally healthy.<br><br>**[page break]**	[randomize order of 1 and 2, display 3 last]<br>1=[Display image of Sun Chips Original multigrain snacks]<br>2=[Display image of Nutella & Go! Cup]<br>3=They’re the same
Identification of healthier product – meal	Click on the product you think is **healthier,** or click “they’re the same” if you think the two products are equally healthy.<br><br>**[page break]**	[randomize order of 1 and 2, display 3 last]<br>1=[Display image of Healthy Choice Café Steamers Chicken Marinara]<br>2=[Display image of Lean Cuisine Turkey with Dressing]<br>3=They’re the same
	**Thinking about healthfulness**	
Prompt	The next questions are about your selections in the shopping task.	
Elaboration-health	When you were selecting foods and beverages in the shopping task, how much did you think about the overall healthfulness of the products?	1=Not at all<br>2=Very little<br>3=Somewhat<br>4=Quite a bit<br>5=A great deal
	**Label Reactions, Perceptions, and Policy Support**	
Programming notes	**[display labels from participants randomly assigned trial arm]**	

© 2025 Grummon AH et al. *JAMA Network Open*.

Construct	Item<br>[programming notes]	Response scale<br>[programming notes]
Notice Nutrition Facts Panel [NFP]	When you were selecting foods and beverages in the shopping task, did you see any Nutrition Facts Panel labels? The Nutrition Facts Panel looks like this.<br><br>**[Display screenshot of how NFP is shown on retailer's website]**<br>**[page break]**	[display vertically]<br>1=Yes<br>0=No<br>2=Not sure
Used NFP	**[display only if marked "yes" to noticing the NFP, otherwise skip]**<br><br>While you were shopping, did you use the Nutrition Facts Panel to decide which foods and beverages to select?	1=Yes<br>0=No
Noticing trial labels	When you were selecting foods and beverages in the shopping task, did you notice any <u>other</u> nutrition labels next to the products (other than the Nutrition Facts Panel)?<br><br>**[page break]**	[display vertically]<br>1=Yes<br>0=No<br>2=Not sure
Used trial label	**[display only if participants marked "yes" to noticing trial labels, otherwise skip]**<br><br>While you were shopping, did you use these other nutrition labels to decide which foods and beverages to select?<br><br>**[page break]**	1=Yes<br>0=No
Prompt & image	**Below are pictures of labels that were on some of the products you saw during the shopping task. The next questions are about this type of label.**<br><br>**[display labels from participants randomly assigned trial arm]**	
Negative emotions – stem	**[Format as matrix; randomize order of emotions]**<br><br>How much do these labels make you feel…	1=Not at all<br>2=Very little<br>3=Somewhat<br>4=Quite a bit<br>5=A great deal
Worry	worried?	
Fear	scared?	
Guilt	guilty?	
Shame	ashamed?	
Sadness	sad? **[page break]**	
Image of label	**[display labels from participants randomly assigned trial arm]**	
Trust information in label	How much do you trust or distrust the information in these labels?	1=Completely distrust<br>2=Somewhat distrust<br>3=Neither trust nor distrust<br>4=Somewhat trust<br>5=Completely trust
Perceived helpfulness	How much do you agree or disagree with the following statement: These labels would help me choose healthier foods and beverages.	1=Strongly disagree<br>2=Somewhat disagree<br>3=Neither disagree nor agree<br>4=Somewhat agree<br>5=Strongly agree

© 2025 Grummon AH et al. *JAMA Network Open*.

Construct	Item<br>[programming notes]	Response scale<br>[programming notes]
Understandability	How much do you agree or disagree with the following statement: It is easy to understand the information in these labels.	1=Strongly disagree<br>2=Somewhat disagree<br>3=Neither disagree nor agree<br>4=Somewhat agree<br>5=Strongly agree
Policy support	Would you oppose or support a policy requiring these labels on foods and beverages?<br><br>**[page break]**	1 = Strongly oppose<br>2 = Somewhat oppose<br>3 = Neither oppose nor support<br>4 = Somewhat support<br>5 = Strongly support
	**Demographics**	
Introduction to demographics	**We are asking the questions in the next section to better understand who completed our survey.**	
Gender	How do you identify?	1=Woman<br>2=Man<br>3=Non-binary<br>4=Prefer to self-describe:_____
Latino or Hispanic ethnicity	Are you of Hispanic, Latino, or Spanish origin?	1=Yes<br>0=No
Race	What is your race? (Check all that apply).	[Check all that apply]<br>1=American Indian or Alaska Native<br>2=Asian<br>3=Black or African American<br>4=Native Hawaiian or Other Pacific Islander<br>5=White<br>6=Another race:________
Education	What is the highest degree or level of school you have completed?	1=Less than high school<br>2=High school graduate (or GED)<br>3=Some college or technical school<br>4=Associate's degree<br>5=Bachelor's degree<br>6=Graduate or professional degree
Prompt	**[Display image of ice cream label modeled after Newest Vital Sign survey].**<br><br>This information is on the back of a container of a pint of ice cream. Please use this information to answer the following questions.<br><br>We don't want you to look up the answers or use a calculator, just give us your best guess.	
Health Literacy – calorie estimation	If you eat the entire container, how many calories will you eat? Type the number of calories in the box.	[Numeric free response]

© 2025 Grummon AH et al. *JAMA Network Open*.

Construct	Item<br>[programming notes]	Response scale<br>[programming notes]
Health Literacy – carbohydrate estimation	If you are allowed to eat 60 grams of carbohydrates as a snack, how much ice cream (in cups) could you have? Type the number of cups in the box.	[Numeric free response]
Health Literacy – saturated fat estimation	Your doctor advises you to reduce the amount of saturated fat in your diet. You usually have 42 g of saturated fat each day, which includes one serving of ice cream. If you stop eating ice cream, how many grams of saturated fat would you be consuming each day? Type the number of grams in the box.	[Numeric free response]
Health Literacy – Daily Value calorie estimation	If you usually eat 2,500 calories in a day, what percentage of your daily value of calories will you be eating if you eat one serving?<br><br>Enter the percentage in the box. For example, enter '50' for 50%.<br><br>**[page break]**	[Numeric free response, restricted to 0-100]
Household size	How many people are in your household, including you?	[# of people [restricted to 1-20, whole numbers]
Income	Which of the following categories best describes your total household income before taxes in the last 12 months?<br><br>**[page break]**	1=Less than $10,000<br>2=$10,000 to $14,999<br>3=$15,000 to $24,999<br>4=$25,000 to $34,999<br>5=$35,000 to $49,999<br>6=$50,000 to $74,999<br>7=$75,000 to $99,999<br>8=$100,000 to $149,999<br>9=$150,000 to $199,999<br>10=$200,000 or more
	**Closure**	
Anything else?	Is there anything else you want to tell us?<br><br>**[page break]**	[Open ended]

© 2025 Grummon AH et al. *JAMA Network Open*.

Construct	Item<br>[programming notes]	Response scale<br>[programming notes]
Debrief	Thank you for taking part in this research study. Because we wanted you to act like you were actually shopping in a store, there was some information about the study that we did not share with you at the beginning of your participation. We would now like to fully explain this research and the survey you just completed.<br><br>All of you will receive your previously agreed upon incentive from the survey company for completing this survey. In addition, in the survey we indicated that 1 in 50 participants would be randomly selected to have the products they chose in the shopping task delivered to them. In actuality, all participants selected for this bonus will receive a $35 gift card, equal to your budget for the shopping task.<br><br>We stated that you would have a chance to receive the products you selected because we wanted you to behave as you normally would when choosing foods and to choose options you would actually want to purchase. This study was conducted by researchers at Stanford University and Harvard Pilgrim Health Care Institute who are interested in the effects of nutrition labels on food and beverage purchases. We will use this study to determine how effective different front-of-package labels are at changing which products people choose.<br><br>Please feel free to contact the investigators, Jason Block and Anna Grummon, with any questions or if you have any concerns about your participation. They can be reached via email at jblock1@mgb.org or agrummon@stanford.edu. If you would like a copy of this debriefing form, please save this page to your computer.	
Closure	You have now finished this survey. Thank you for your participation!	

© 2025 Grummon AH et al. *JAMA Network Open*.

eTable 4. Comparison of Characteristics of the Study Sample (5636 US Adults) to National Estimates

	Study sample %	National estimate %
Age		
18-29 years	21%	20%
30-44 years	47%	26%
45-59 years	23%	23%
60 years or older	10%	31%
Gender		
Woman	60%	51%
Man	38%	49%
Non-binary or another gender	2%	NA
Latino(a) or Hispanic	10%	18%
Race		
American Indian or Alaska Native	1%	1%
Asian, Native Hawaiian, or Pacific Islander	6%	6%
Black or African American	11%	12%
White	76%	63%
Another race or multiracial	6%	18%
Education		
High school diploma or less	11%	37%
Some college	21%	20%
College graduate or associate degree	51%	29%
Graduate degree	16%	13%
Household income, annual		
$0 to $24,999	13%	14%
$25,000 to $49,999	21%	17%
$50,000 to $74,999	21%	16%
$75,000 or more	44%	53%

[a]National estimates for age, gender, race and ethnicity, and education are survey-weighted estimates among adults (ages 18-years and older) in the 2023 American Community Survey (ACS) 1-year Public Use Microdata Sample (PUMS).[1] National estimate of % of people identifying as non-binary or another gender is listed as "NA" because the ACS did not include an option for people to identify as non-binary or another gender. National estimate of % of people identifying as Middle Eastern or North African is listed as "NA" because the ACS did not provide data on the proportion of respondents identifying in this category. National estimates for income are from the Current Population Survey, 2023.[2]

© 2025 Grummon AH et al. *JAMA Network Open*.

eTable 5. Healthfulness and Nutrient Content of and Spending on Food and Beverage Selections, by Front-of-Package Labeling System (5636 US Adults)

Outcomes	Positive Mean	(SE)	Nutrition Info Mean	(SE)	High In Mean	(SE)	Positive plus Nutrition Info Mean	(SE)	Positive plus High In Mean	(SE)	Spectrum Mean	(SE)
**Outcomes to increase**												
Healthfulness (Ofcom score, primary outcome)	58.5	(.3)	58.5	(.3)	58.9	(.3)	59.0	(.3)	59.1	(.3)	60.9	(.3)
Healthfulness (Guiding Stars score)	-1.6	(.1)	-2.0	(.1)	-1.5	(.1)	-1.8	(.1)	-1.4	(.1)	-.8	(.1)
Fiber density, g per 100g	2.8	(.1)	2.7	(.1)	2.8	(.1)	2.6	(.1)	2.7	(.1)	3.0	(.1)
Fiber per serving, g	1.4	(.03)	1.4	(.03)	1.4	(.03)	1.3	(.03)	1.4	(.03)	1.6	(.03)
Total fiber, g	81.6	(2.8)	81.0	(2.8)	84.0	(2.8)	78.5	(2.8)	83.4	(2.8)	89.4	(2.8)
Protein density, g per 100g	6.4	(.1)	6.3	(.1)	6.7	(.1)	6.3	(.1)	6.4	(.1)	6.7	(.1)
Protein per serving, g	3.6	(.1)	3.8	(.1)	3.8	(.1)	3.7	(.1)	3.7	(.1)	4.0	(.1)
Total protein, g	217.8	(7.3)	219.6	(7.3)	224.6	(7.3)	220.5	(7.3)	228.2	(7.3)	223.8	(7.3)
**Outcomes to decrease**												
Number of items high in ≥1 nutrient of concern	3.7	(.1)	3.8	(.1)	3.3	(.1)	3.6	(.1)	3.2	(.1)	3.3	(.1)
Calorie density, kcal per 100g	265.6	(3.2)	260.4	(3.2)	259.6	(3.2)	255.0	(3.2)	257.8	(3.2)	250.6	(3.2)
Calories per serving, kcal	129.2	(1.4)	131.7	(1.5)	127.3	(1.4)	128.3	(1.4)	127.6	(1.5)	130.4	(1.4)
Total calories, kcal	8104.4	(185.1)	8116.5	(185.2)	7926.1	(184.2)	7920.4	(184.8)	8100.5	(185.3)	7608.5	(184.4)
Sugar density, g per 100g	8.3	(.2)	8.5	(.2)	7.9	(.2)	8.3	(.2)	7.9	(.2)	7.8	(.2)
Sugar per serving, g	5.9	(.2)	6.0	(.2)	5.4	(.2)	5.7	(.2)	5.6	(.2)	5.7	(.2)
Total sugar, g	377.2	(11.5)	371.0	(11.5)	343.0	(11.4)	360.7	(11.5)	363.3	(11.5)	345.2	(11.4)
Saturated fat density, g per 100g	2.1	(.1)	2.0	(.1)	2.2	(.1)	2.0	(.1)	2.0	(.1)	1.7	(.1)
Saturated fat per serving, g	1.1	(.0)	1.1	(.0)	1.1	(.0)	1.0	(.0)	1.0	(.0)	1.0	(.0)
Total saturated fat, g	65.5	(3.7)	65.9	(3.7)	65.5	(3.7)	65.3	(3.7)	67.0	(3.7)	56.3	(3.7)
Sodium density, mg per 100g	382.0	(7.8)	392.1	(7.8)	395.0	(7.7)	369.8	(7.8)	372.2	(7.8)	346.1	(7.7)
Sodium per serving, mg	206.2	(3.7)	219.1	(3.7)	208.7	(3.7)	213.1	(3.7)	202.9	(3.7)	198.8	(3.7)
Total sodium, mg	11865.2	(222.0)	12118.6	(222.3)	11884.1	(221.0)	11759.2	(221.7)	11713.5	(222.4)	10675.8	(221.2)
**Neutral outcomes**												

© 2025 Grummon AH et al. *JAMA Network Open*.

Number of items selected	8.5	(.1)	8.7	(.1)	8.5	(.1)	8.5	(.1)	8.5	(.1)	8.4	(.1)
Spending, USD ($)	31.7	(.2)	31.7	(.2)	31.6	(.2)	31.6	(.2)	31.6	(.2)	31.3	(.2)

Table shows estimated means and SEs from regression models.

© 2025 Grummon AH et al. *JAMA Network Open*.

eTable 6. Impact of Front-of-Package Labeling Systems on Healthfulness of Food and Beverage Purchases, by Nutrition Literacy, Household Income, and Educational Attainment (5636 US Adults)

	Difference in healthfulness vs. positive labels										
	Nutrition Info		High In		Positive plus Nutrition Info		Positive plus High In		Spectrum		*p* for interaction[a]
**Characteristic**	**ADE**	**(95% CI)**	**ADE**	**(95% CI)**	**ADE**	**(95% CI)**	**ADE**	**(95% CI)**	**ADE**	**(95% CI)**	
Nutrition literacy, mean=3.29, SD=1.03											
Mean – 1 SD	.38	(-.71, 1.48)	.94	(-.14, 2.02)	.79	(-.30, 1.87)	.88	(-.19, 1.96)	2.69	(1.61, 3.78)	
Mean	-.02	(-.78, .74)	.39	(-.36, 1.15)	.48	(-.28, 1.24)	.61	(-.15, 1.36)	2.44	(1.68, 3.20)	0.82
Mean + 1 SD	-.43	(-1.49, .63)	-.15	(-1.22, .91)	.17	(-.90, 1.25)	.33	(-.75, 1.41)	2.19	(1.13, 3.25)	
Household income											
$0 to $24,999	.76	(-1.38, 2.90)	.18	(-1.96, 2.33)	.02	(-2.16, 2.20)	.81	(-1.35, 2.98)	2.27	(.08, 4.47)	
$25,000 to $49,999	.40	(-1.23, 2.03)	.79	(-.86, 2.44)	1.56	(-.08, 3.19)	.99	(-.61, 2.59)	3.38	(1.72, 5.04)	
$50,000 to $74,999	-1.71	(-3.39, -.02)	-.91	(-2.52, .70)	-.02	(-1.65, 1.62)	-.35	(-2.01, 1.31)	.05	(-1.59, 1.68)	0.29
$75,000 or more	.28	(-.85, 1.42)	.83	(-.31, 1.98)	.26	(-.88, 1.40)	.68	(-.47, 1.83)	3.12	(2.00, 4.25)	
Educational attainment											
High school diploma or less	2.45	(.22, 4.68)	2.06	(-.19, 4.32)	2.71	(.55, 4.86)	1.71	(-.52, 3.94)	2.97	(.75, 5.18)	
Some college	-.40	(-2.10, 1.29)	.41	(-1.33, 2.15)	.03	(-1.70, 1.76)	.37	(-1.27, 2.01)	2.23	(.58, 3.88)	
College graduate or associate degree	-.15	(-1.22, .91)	.23	(-.81, 1.27)	.34	(-.70, 1.39)	.71	(-.35, 1.77)	2.89	(1.82, 3.96)	0.64
Graduate degree	-1.15	(-2.97, .67)	-.73	(-2.57, 1.12)	-.28	(-2.18, 1.62)	-.48	(-2.44, 1.48)	.93	(-.92, 2.78)	

Abbreviations: ADE, average differential effect; CI, confidence interval.

[a] *p*-value for joint significance of the interaction terms.

© 2025 Grummon AH et al. *JAMA Network Open*.

eTable 7. Comparison of Effects of Novel Front-of-Package Labeling Systems on Healthfulness of Food and Beverage Purchases (5636 US Adults)

Contrast	Difference in healthfulness		
	ADE (95% CI)		*p*-value
Nutrition Info			
vs. High In	-.39	(-1.15 , .37)	.31
vs. positive plus Nutrition Info	-.48	(-1.23 , .28)	.22
vs. positive plus High In	-.58	(-1.34 , .18)	.14
vs. spectrum	**-2.45**	**(-3.21 , -1.69)**	**<.001**
High In			
vs. positive plus Nutrition Info	-.08	(-.84 , .67)	.83
vs. positive plus High In	-.19	(-.95 , .57)	.63
vs. spectrum	**-2.06**	**(-2.82 , -1.30)**	**<.001**
Positive plus Nutrition Info			
vs. positive plus High In	-.10	(-.86 , .65)	.79
vs. spectrum	**-1.98**	**(-2.73 , -1.22)**	**<.001**
Positive plus High In			
vs. spectrum	**-1.87**	**(-2.63 , -1.11)**	**<.001**

Abbreviations: ADE, average differential effect; CI, confidence interval.
Note. **Bolded** cells are statistically significant, *p*<.05.

© 2025 Grummon AH et al. *JAMA Network Open*.

eTable 8. Impact of Front-of-Package Labeling Systems on Secondary Selection Outcomes (5636 US Adults)

Outcomes	**Difference in outcome vs. positive labels**									
	**Nutrition Info**		**High In**		**Positive plus Nutrition Info**		**Positive plus High In**		**Spectrum**	
	**ADE (95% CI)**		**ADE (95% CI)**		**ADE (95% CI)**		**ADE (95% CI)**		**ADE (95% CI)**	
**Outcomes to increase**										
Fiber per serving, g	-.01	(-.10, .07)	.03	(-.06, .11)	-.03	(-.12, .06)	.02	(-.06, .11)	**.21**	**(.13, .30)**
Total fiber, g	-.68	(-8.42, 7.06)	2.32	(-5.40, 10.04)	-3.18	(-10.91, 4.55)	1.77	(-5.98, 9.51)	**7.75**	**(.03, 15.47)**
Protein per serving, g	.12	(-.08, .32)	.16	(-.04, .36)	.08	(-.12, .28)	.10	(-.10, .30)	**.35**	**(.15, .55)**
Total protein, g	1.74	(-18.47, 21.95)	6.80	(-13.35, 26.96)	2.68	(-17.51, 22.86)	10.35	(-9.86, 30.57)	5.92	(-14.24, 26.08)
**Outcomes to decrease**										
Calories per serving, kcal	2.50	(-1.52, 6.52)	-1.97	(-5.98, 2.04)	-.97	(-4.98, 3.05)	-1.63	(-5.65, 2.39)	1.21	(-2.80, 5.22)
Total calories, kcal	12.16	(-501.15, 525.47)	-178.24	(-690.06, 333.57)	-183.95	(-696.58, 328.67)	-3.85	(-517.30, 509.59)	-495.83	(-1007.91, 16.26)
Sugar per serving, g	.10	(-.36, .57)	**-.49**	**(-.95, -.02)**	-.17	(-.64, .29)	-.23	(-.70, .23)	-.18	(-.64, .29)
Total sugar, g	-6.28	(-38.10, 25.55)	**-34.28**	**(-66.01, -2.55)**	-16.57	(-48.35, 15.21)	-13.93	(-45.76, 17.90)	**-32.00**	**(-63.75, -.25)**
Saturated fat per serving, g	.06	(-.03, .14)	-.01	(-.10, .07)	-.02	(-.10, .06)	-.06	(-.15, .02)	**-.11**	**(-.20, -.03)**
Total saturated fat, g	.48	(-9.76, 10.73)	.01	(-10.20, 10.23)	-.11	(-10.34, 10.12)	1.56	(-8.69, 11.81)	-9.16	(-19.38, 1.06)
Sodium per serving, mg	**12.90**	**(2.57, 23.24)**	2.50	(-7.81, 12.81)	6.93	(-3.39, 17.26)	-3.29	(-13.63, 7.05)	-7.39	(-17.71, 2.92)
Total sodium, mg	253.37	(-362.57, 869.31)	18.88	(-595.26, 633.02)	-106.02	(-721.14, 509.09)	-151.71	(-767.81, 464.39)	**-1189.38**	**(-1803.85, -574.92)**

Abbreviations: ADE, average differential effect; CI, confidence interval.
Note. **Bolded** cells are statistically significant, $p<.05$.

© 2025 Grummon AH et al. *JAMA Network Open*.

eTable 9. Impact of Front-of-Package Labeling Systems on Consumer Understanding, Label Reactions and Perceptions, and Public Support (5636 US Adults)

	Differences vs. positive labels									
	Nutrition Info		High In		Positive plus Nutrition Info		Positive plus High In		Spectrum	
Outcomes	ADE (95% CI)		ADE (95% CI)		ADE (95% CI)		ADE (95% CI)		ADE (95% CI)	
**Consumer understanding**										
Correctly identified healthier product	**.19**	**(.17, .22)**	**.05**	**(.03, .08)**	**.19**	**(.16, .21)**	**.12**	**(.10, .15)**	**.12**	**(.09, .14)**
**Label reactions and perceptions**										
Noticed label	**.07**	**(.02, .11)**	**.21**	**(.17, .25)**	**.12**	**(.07, .16)**	**.24**	**(.20, .29)**	**.10**	**(.05, .14)**
Used label when shopping	.04	(-.04, .12)	**.13**	**(.06, .21)**	.03	(-.04, .11)	**.13**	**(.06, .20)**	**.08**	**(.002, .16)**
Thinking about health	-.07	(-.17, .03)	**.12**	**(.02, .22)**	-.002	(-.10, .10)	**.16**	**(.06, .26)**	**.15**	**(.05, .25)**
Negative emotions	**.45**	**(.38, .52)**	**.67**	**(.61, .74)**	**.28**	**(.21, .34)**	**.42**	**(.36, .49)**	**.47**	**(.40, .54)**
Perceived helpfulness	**.44**	**(.35 , .53)**	**.40**	**(.31 , .49)**	**.38**	**(.29 , .46)**	**.37**	**(.28 , .45)**	**.17**	**(.08 , .26)**
Perceived understandability	**.47**	**(.39, .55)**	**.48**	**(.40, .57)**	**.44**	**(.36, .52)**	**.34**	**(.25, .42)**	**.13**	**(.05, .21)**
Perceived trustworthiness	**.46**	**(.39, .53)**	**.60**	**(.53, .68)**	**.37**	**(.29, .44)**	**.35**	**(.28, .43)**	.06	(-.02, .13)
**Public support**										
Support for requiring this labeling system	.07	(-.02, .15)	**.12**	**(.03, .20)**	.02	(-.07, .11)	**.10**	**(.02, .19)**	-.05	(-.13, .04)

Abbreviations: ADE, average differential effect; CI, confidence interval.
Note. **Bolded** cells are statistically significant, p<.05.

© 2025 Grummon AH et al. *JAMA Network Open*.

eReferences

1. United States Food and Drug Administration. Code of Federal Regulations Title 21, Volume 2, Subpart D - Specific Requirements for Nutrient Content Claims. Code of Federal Regulations. Published December 22, 2023. Accessed March 26, 2024. https://www.accessdata.fda.gov/scripts/cdrh/cfdocs/cfcfr/CFRSearch.cfm?CFRPart=101&showFR=1&subpartNode=21:2.0.1.1.2.4

2. US Food and Drug Administration. Daily Value on the Nutrition and Supplement Facts Labels. FDA. Published March 13, 2024. Accessed March 29, 2024. https://www.fda.gov/food/nutrition-facts-label/daily-value-nutrition-and-supplement-facts-labels

3. Guiding Stars LLC. How It Works. Guiding Stars. Published 2024. Accessed March 26, 2024. https://guidingstars.com/what-is-guiding-stars/

© 2025 Grummon AH et al. *JAMA Network Open*.
