# Supplementary material for: Nutrition Info and Other Front-of-Package Labels and Simulated Food and Beverage Purchases: A Randomized Clinical Trial
Source: JAMA Netw Open. 2025 Oct 17;8(10):e2537389. doi: 10.1001/jamanetworkopen.2025.37389 (PMC12534843; doi:10.1001/jamanetworkopen.2025.37389)
Supplement: Supplement 3. — Data Sharing Statement [file jamanetwopen-e2537389-s003.pdf]

Data Sharing Statement

Grummon. Nutrition Info and Other Front-of-Package Labels and Simulated Food and Beverage Purchases. *JAMA Netw Open*. Published October 17, 2025. doi:10.1001/jamanetworkopen.2025.37389

Data
Additional Information: ClinicalTrials.gov, https://clinicaltrials.gov/study/NCT06516627?term=nutrition%20labels&rank=1, #NCT06516627
Data available: Yes
Data types: Deidentified participant data
How to access data: We will post the data publicly after publication at https://github.com/annagrummon/NutritionInfoFOPLs_JNO2025.
When available: With publication

Supporting Documents
Document types: None

Additional Information
Who can access the data: Anyone can download
Types of analyses: For any purpose
Mechanisms of data availability: Post publicly
Any additional restrictions: NA
